# Supplementary material for: Scattered tree death contributes to substantial forest loss in California
Source: Nat Commun. 2024 Jan 20;15:641. doi: 10.1038/s41467-024-44991-z (PMC10799937; doi:10.1038/s41467-024-44991-z)
Supplement: Supplementary file 1 — Supplementary Information [file 41467_2024_44991_MOESM1_ESM.pdf]

Supplementary Information

Scattered tree death contributes to substantial forest loss in California

Yan Cheng[1,*], Stefan Oehmcke[2], Martin Brandt[1], Lisa Rosenthal[3], Adrian Das[3], Anton Vrieling[4], Sassan Saatchi[5,6], Fabien Wagner[5,6], Maurice Mugabowindekwe[1], Wim Verbruggen[1], Claus Beier[1], Stephanie Horion[1,*]

[1]Department of Geosciences and Natural Resource Management, University of Copenhagen, Copenhagen, Denmark
[2]Department of Computer Science, University of Copenhagen, Copenhagen, Denmark
[3]US Geological Survey, Western Ecological Research Center, Three Rivers, Sequoia and Kings Canyon Field Station, California, USA
[4]Faculty of Geo-Information Science and Earth Observation (ITC), University of Twente, Enschede, the Netherlands
[5]University of California, Los Angeles, USA
[6]Jet Propulsion Laboratory, California Institute of Technology, Pasadena, USA

*Correspondence to yach@ign.ku.dk and smh@ign.ku.dk

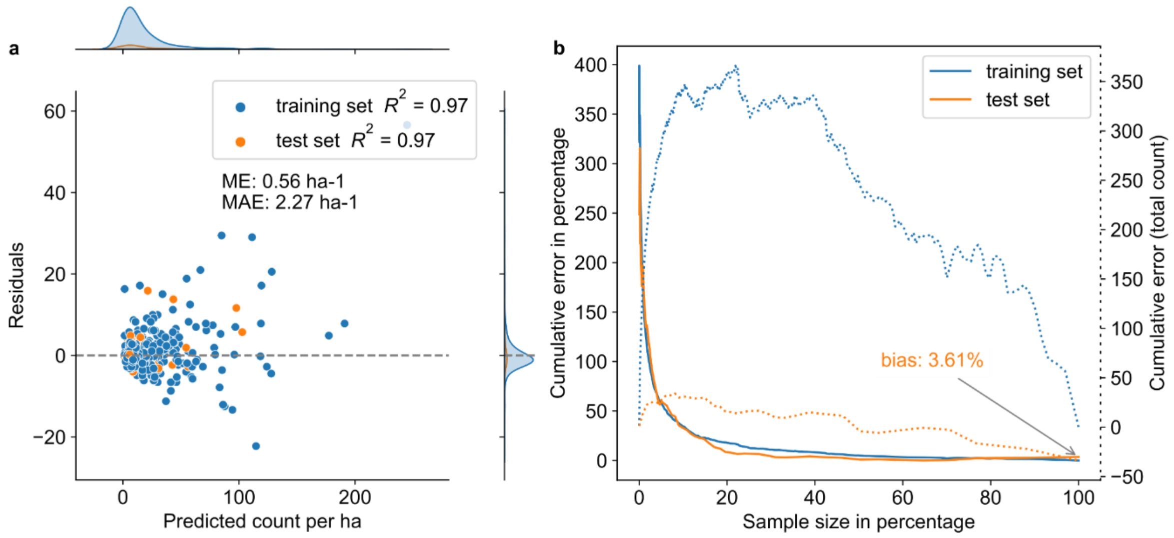

Supplementary Fig. 1: Model evaluation against manually labelled dead trees at the plot level. a, residual plot for the count of dead trees per ha (Methods). The x-axis represents the predicted count per ha (Methods), while the y-axis is the residuals indicating the difference between the predicted count and actual count per ha. The training set (i.e. training and evaluation set; n=567) and test set (n=60) are denoted in blue and orange colour, respectively. Each point represents a 256 × 256 pixel area on NAIP image, which is equivalent to a 2.36 ha plot on the ground. b, cumulative error in percent (solid lines) and cumulative error (total count; dotted lines) as a function of sample size (in percent).. The blue lines and orange lines denote the training set and test set, respectively. The bias, MAE, and ME are calculated following Equation (5), (1), (3).

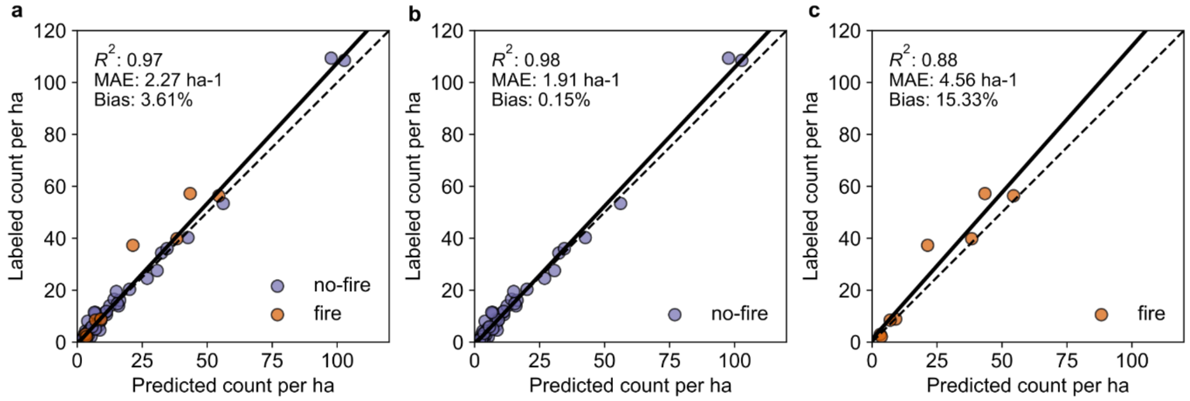

Supplementary Fig. 2: Model evaluation for no-fire and fire-impacted areas at the plot level. a, fire and no-fire plots (n=60). b, no fire plots (n=52). c, fire plots (n=8). Each point represents a 256 × 256 pixel area on NAIP image, which is equivalent to a 2.36 ha plot on the ground. The bias are calculated following Equation (5).

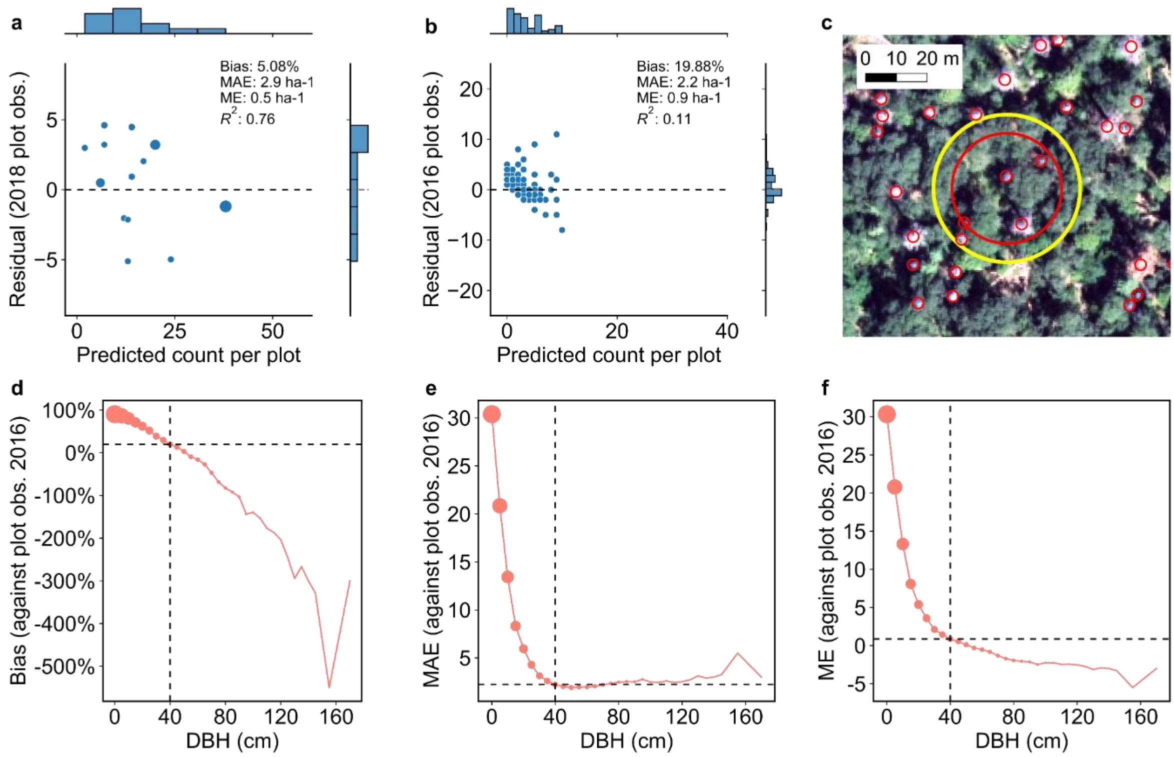

Supplementary Fig. 3: Evaluation against ground observations of dead tree count at the plot level. a, comparison to dead tree counts for 13 plots visited in 2018[73] (Methods). Each dot represents a plot and is sized proportionally to the plot size between 0.93 and 3.35 ha. The x-axis is the predicted count per plot aggregated from the individual dead tree map without count bias correction. The y-axis represents the residuals which is the difference between ground observations and model predictions. The negative residuals represent potential underestimations of dead tree count in the predictions. Residuals are converted into count per ha based on the plot size. Panel b-f are the comparison to 75 0.1 ha-large plots visited in 2016[13] (Methods). Panel b is the residual plot of dead tree count per plot for dead trees ≥40 cm in DBH. Panel c illustrates the plot design. The big red circle in the middle represents the actual plot while the yellow circle represents the plot with a 6 m buffer. The small red circles indicate the centroid of each dead tree detected by the model. The background is an NAIP image with image stretch applied. Panel d - f illustrate three error metrics (i.e. bias, MAE, and ME) as a function of DBH cutoffs that were used to filter ground observations (Methods). The dot size is proportional to the number of plots after DBH-filtering. The largest number of plots is 87 plots when no DBH filtering is applied (i.e. DBH value on the x-axis is 0). The bias is calculated following Equation (5). The MAE and ME are calculated following Equation (1) and (3).

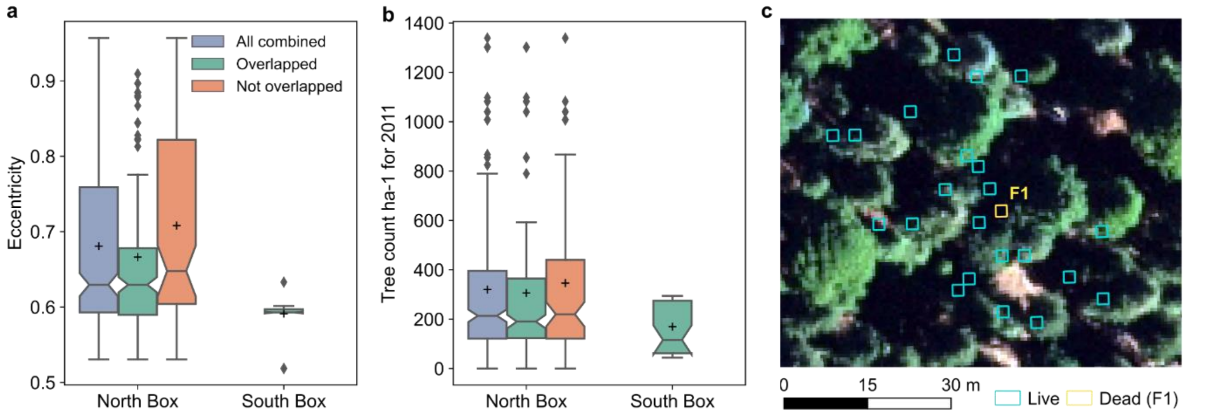

Supplementary Fig. 4: Off-nadir view-related omission errors in the tree-level accuracy assessment against ground observations in 2020. Panel a and b are boxplots of eccentricities (a) and tree count ha-1 for 2011 (b) for dead tree locations inside the north box (n=294) and south box (n=32) of the field survey 2020 (MCVNB2020 and SMSB2020). Larger eccentricities indicate larger image distortion due to off-nadir viewing angles. c, examples of an invisible dead tree (yellow box) from NAIP images due to off-nadir view angles-caused image distortion. The label besides the yellow box refers to the decay class of a dead tree observed in the field, with F1 characterising dead trees that retain less than one-third of its dead foliage. In panel a and b, the blue, green, and orange boxes represent the distribution of eccentricities and tree density for all dead trees, dead trees overlapped, and not overlapped with the predictions, respectively. All dead trees in the south box overlapped with the predictions, therefore, there is only one green box. The boxes represent the interquartile range (IQR) of tree mortality. The whiskers represent 1.5 times the IQR. The black lines inside the boxes represent the medians, whereas the mean values are indicated by crosses. The notches inside boxes represent the 95% confidence intervals for the medians. The black cross inside each box represents the mean value.

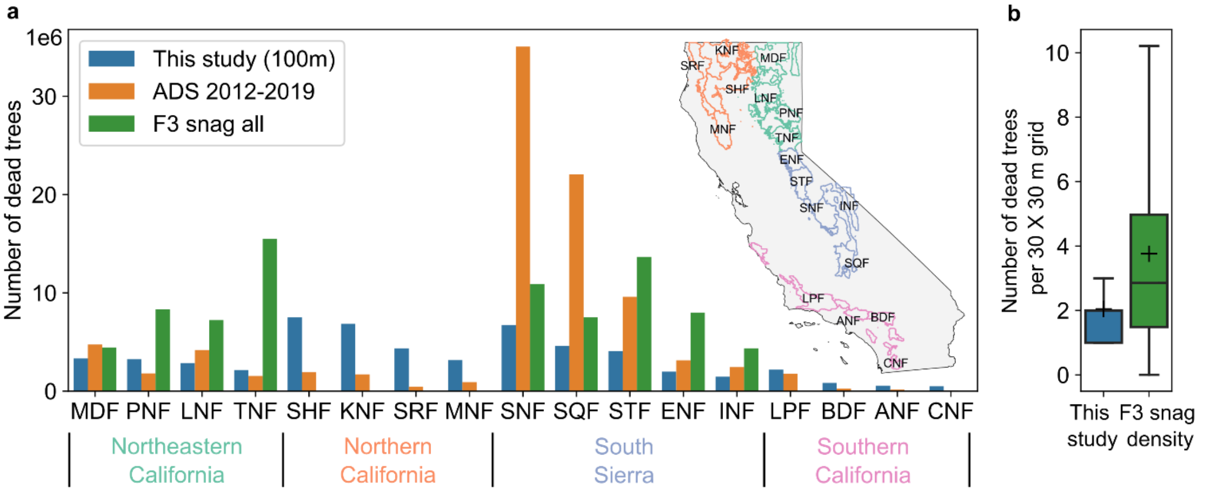

Supplementary Fig. 5: Comparison to current reports of tree mortality by national forests in California. a, total number of dead trees for 17 national forests from this study and ADS[10] and nine national forests from F3 model-based snag density map[66]. b, boxplots of the number of dead trees within 30 × 30 m grids for areas where dead trees were detected in this study. The boxes represent the interquartile range (IQR) of the number of dead trees within a 30 × 30 m grid. The whiskers represent 1.5 times the IQR. The black line inside each box represents the median. The black cross inside each box represents the mean value.

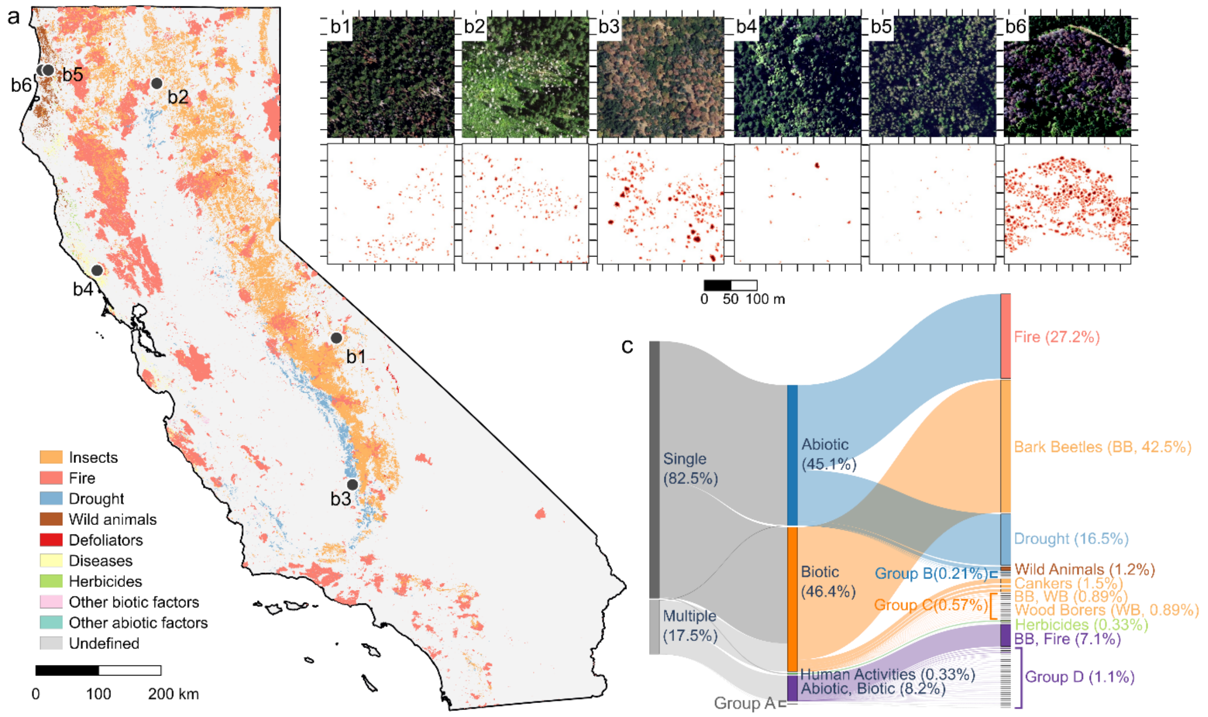

Supplementary Fig. 6: Damage agent attribution based on ADS database and fire perimeters from 2012-2020. a, spatial distribution of the most recent and dominant damage agents (Level 2 category). b, examples of tree mortality related to six dominant damage agents (Level 2 category): b1, Insects (e.g., Bark Beetles), b2, Fire, b3, Drought, b4, Diseases, b5, Wild animal (e.g., Wild boars), b6, Human activity (e.g., Herbicides). Each panel consists of two items. The top shows the true colour NAIP image, and the bottom shows the dead tree predictions. The black lines on the edge of each image represent 30 × 30 m grids. The geolocation of each sample area is indicated on the left-hand map. c, percentage of total number of dead trees associated with each type of damage agent (Level 1 and Level 3 category). Group A consists of two categories of multiple damage agents: Human Activities and Abiotic Agents (0.0011%) and Human Activities and Biotic Agents (0.00014%). The detailed information for Group B, C, and D is listed in Supplementary Table 9.

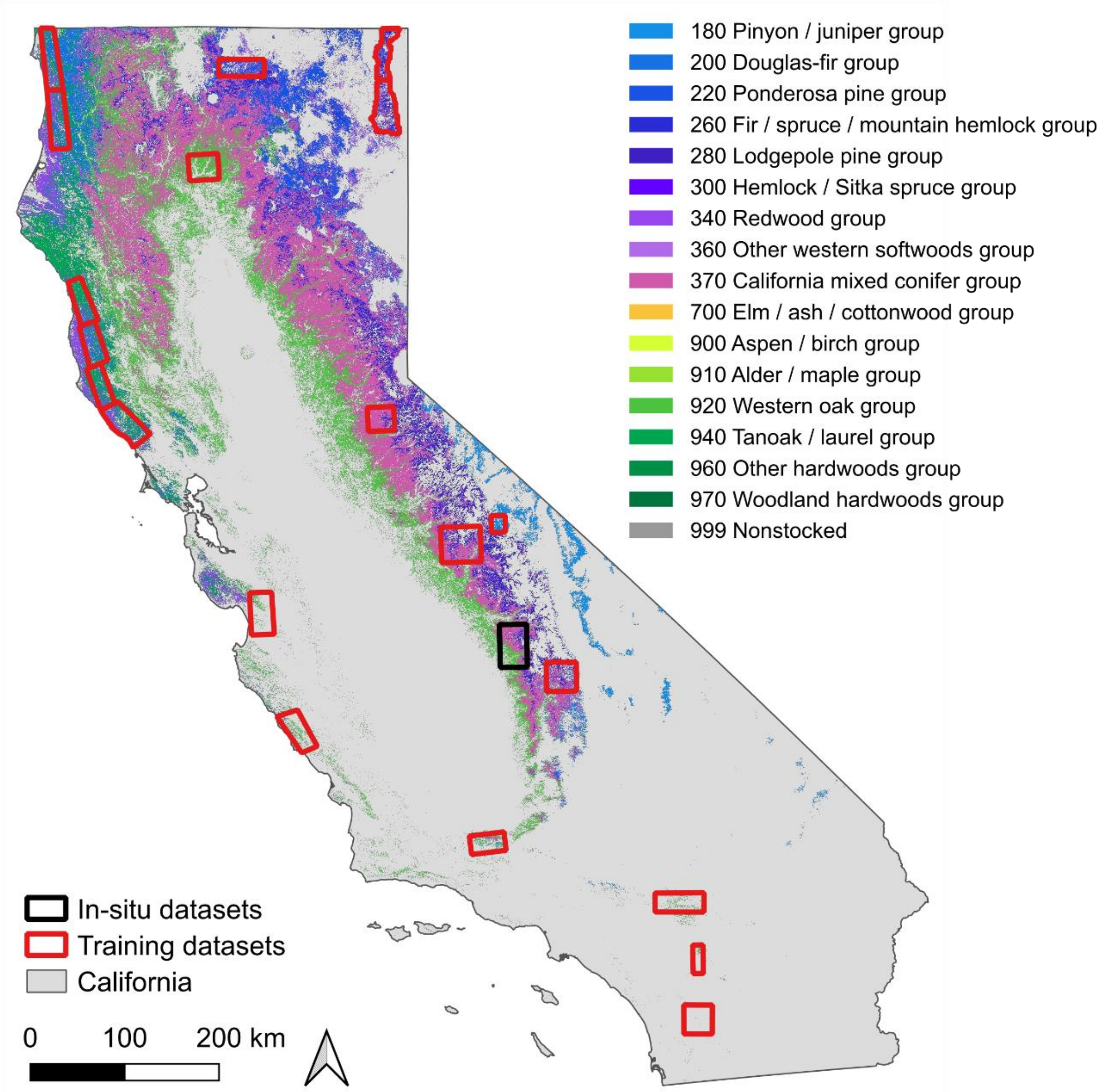

Supplementary Fig. 7: Training sample distribution and forest type map. The red boxes indicate the bounding boxes of training and test patches (overlapping with preselected areas of interest for ADS survey 2020[10]). The black box indicates the location of ground observations. The background map shows the Forest Type Groups of the Continental United States[23].

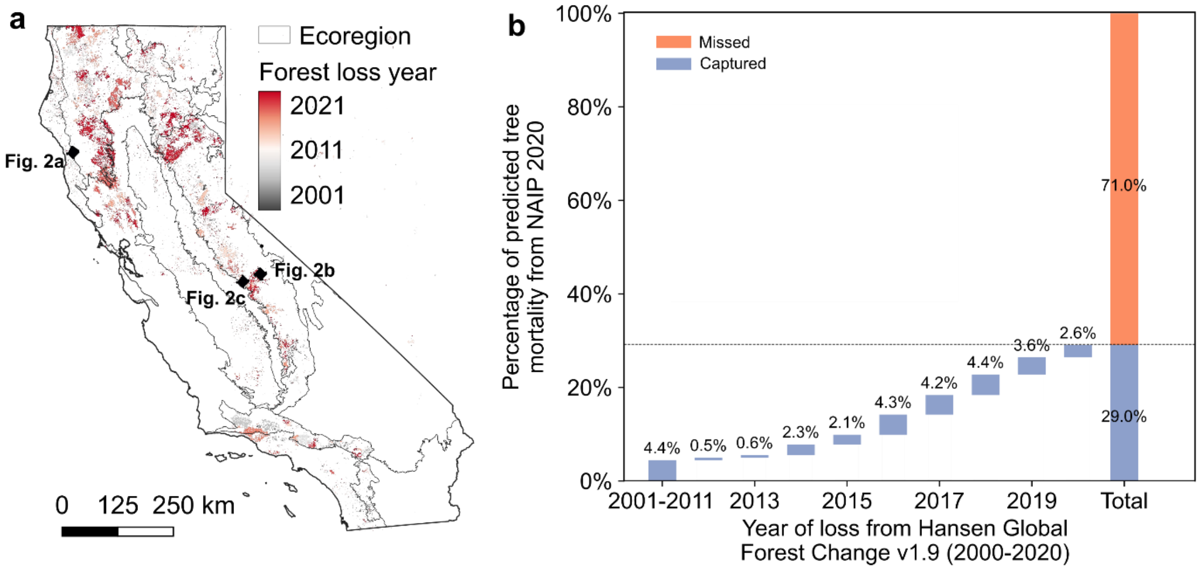

Supplementary Fig. 8: Comparison to Landsat-based Global Forest Change v1.9 (2000-2020) (referred to as forest loss data hereafter). **a,** map of forest loss year. The black diamond shapes represent the geolocations of Fig. 2a-c in the main text. **b**, percentage of dead trees detected in this study grouped by forest loss years and by the number of dead trees within a 30 × 30 m grid. The blue bars indicate the proportion of dead trees detected that overlaps with forest loss data in different years. The orange bar represents the percentage of dead trees detected in this study that was not included in the forest loss data.

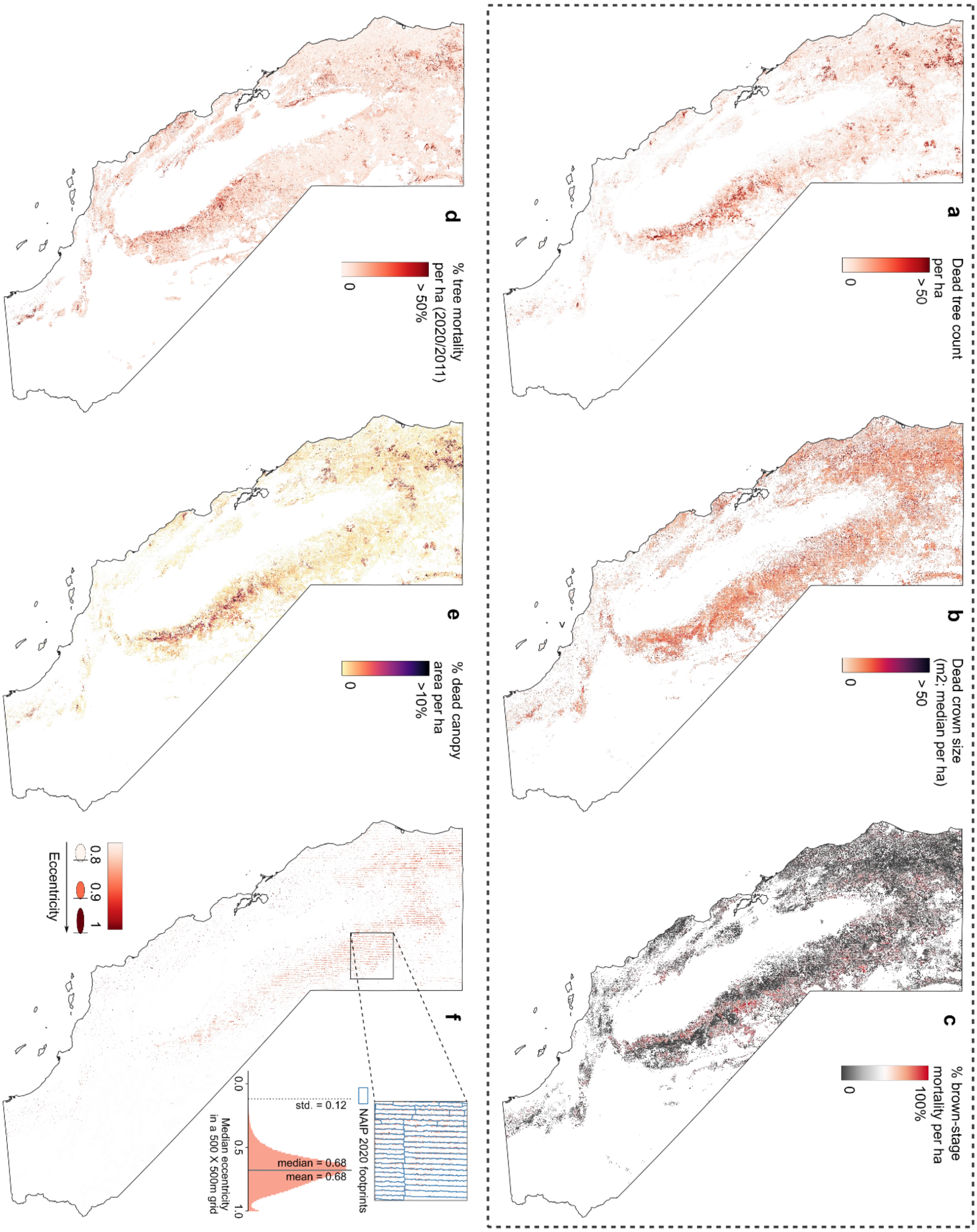

Supplementary Fig. 9: Structural metrics and mortality stages of dead trees derived from the individual dead tree map. Panel a-e are dead tree metrics aggregated from individual dead trees within hectare grids: a, number of dead trees per ha, b, median of dead tree crown sizes per ha, c, percentage of brown-stage mortality, d, percentage of dead trees using the number of trees for 2011 as the initial state of live tree count, e, percentage of dead canopy area per ha, Panel f is the quantification of geometric distortion caused by off-nadir view angles. The map shows the median eccentricity of dead tree crowns with more than 50 NAIP pixels within 500 × 500 m grids. The top right corner of this panel illustrates the alignment between areas with high eccentricity (>0.8) and NAIP 2020 footprints. The maps in the dotted line box (i.e. a-c) are the ones used to synthesise Fig. 2 in the main text.

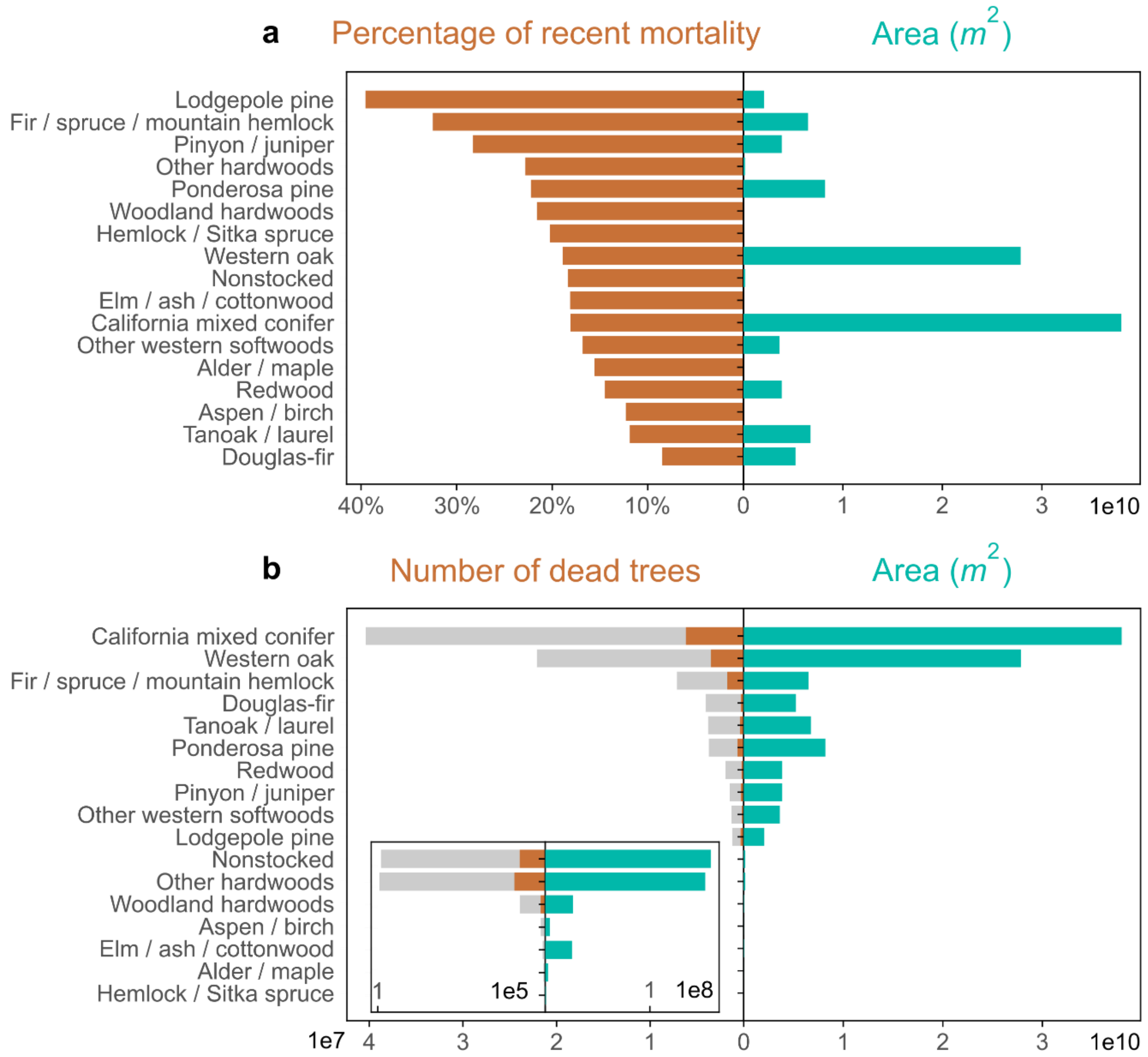

Supplementary Fig. 10: Brown- and grey-stage mortality by forest type groups. a, percentage of brown-stage mortality in descending order. b, count of dead trees in brown- and grey-stage in descending order of the total number of dead trees. The embedded chart in b on the left bottom is a zoom-in view of forest-type groups with relatively low numbers of dead trees, from Nonstocked to Hemlock / Sitka spruce group. For both a and b, brown and grey bars represent brown- and grey-stage mortality, respectively. Green bars on the right panels represent the spatial coverage (m^2) of each forest type group.

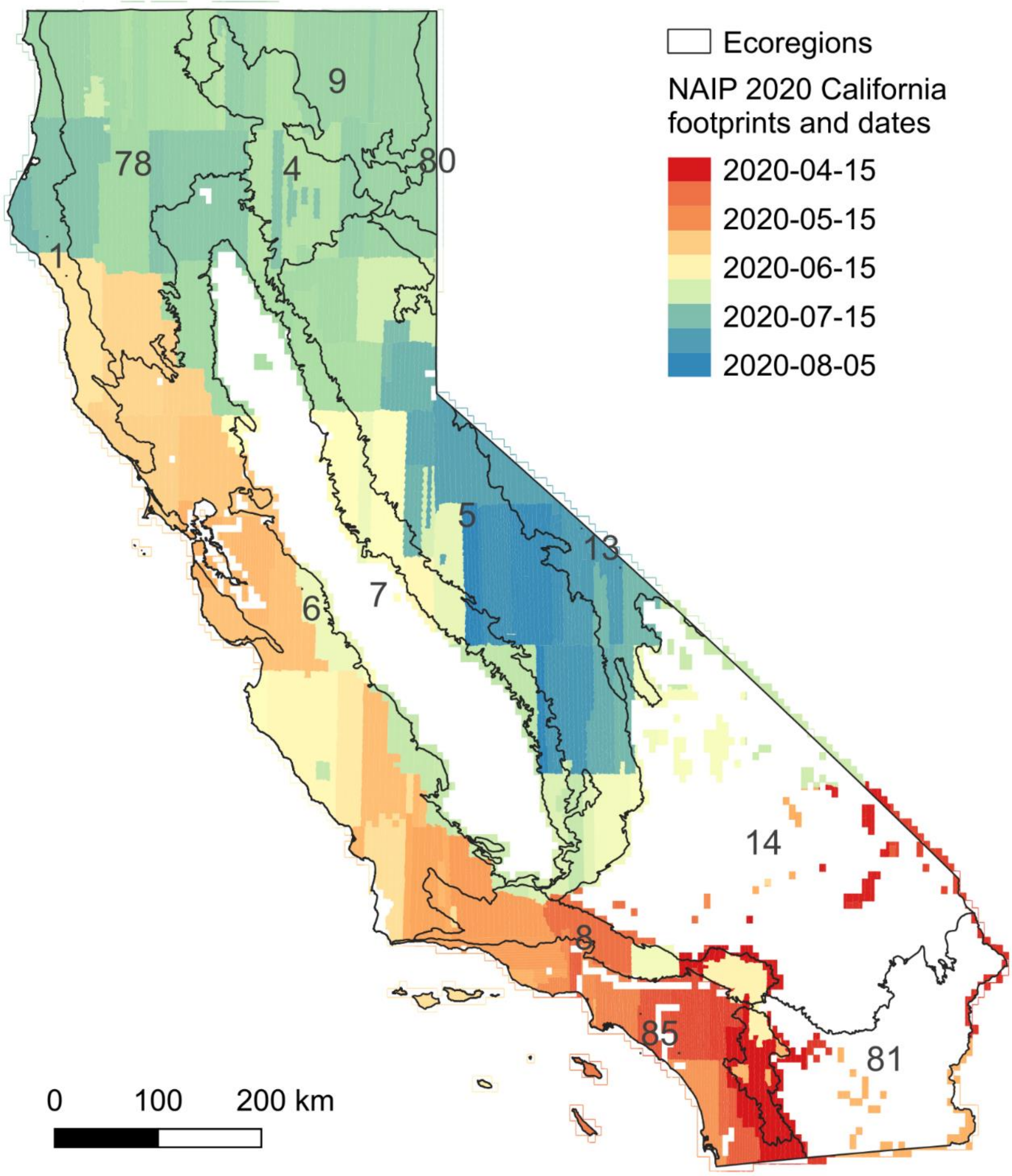

Supplementary Fig. 11: Footprints and acquisition dates of NAIP images used in this study. The acquisition dates range from red (2020-04-15) to yellow (2020-06-15) to blue (2020-08-05). The black polygons represent the boundaries of level-3 ecoregions in California[74]. The numbers are the unique codes of each level-3 ecoregion: 1-Coast Range, 4-Cascades, 5-Sierra Nevada, 6-Central California Foothills and Coastal Mountains, 7-Central California Valley, 8-Southern California Mountains, 9-Eastern Cascades Slopes and Foothills, 13-Central Basin and Range, 14-Mojave Basin and Range, 78-Klamath Mountains/California High North Coast Range, 80-Northern Basin and Range, 81-Sonoran Basin and Range, 85-Southern California/Northern Baja Coast.

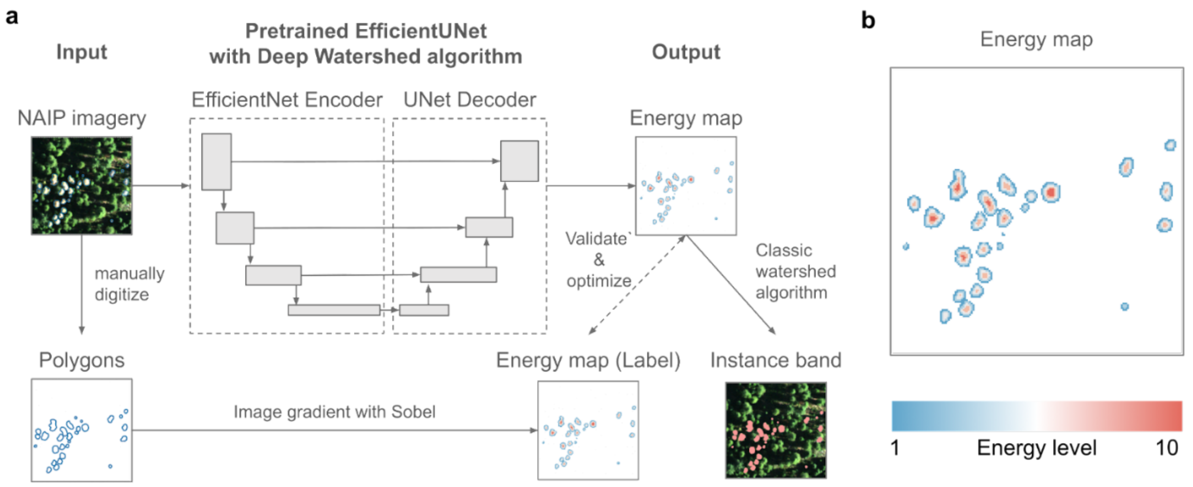

Supplementary Fig. 12: Schematic of pre-trained EfficientUNet with the deep watershed algorithm for individual dead tree detection from NAIP images. a, model architecture. b, illustration of the energy map.

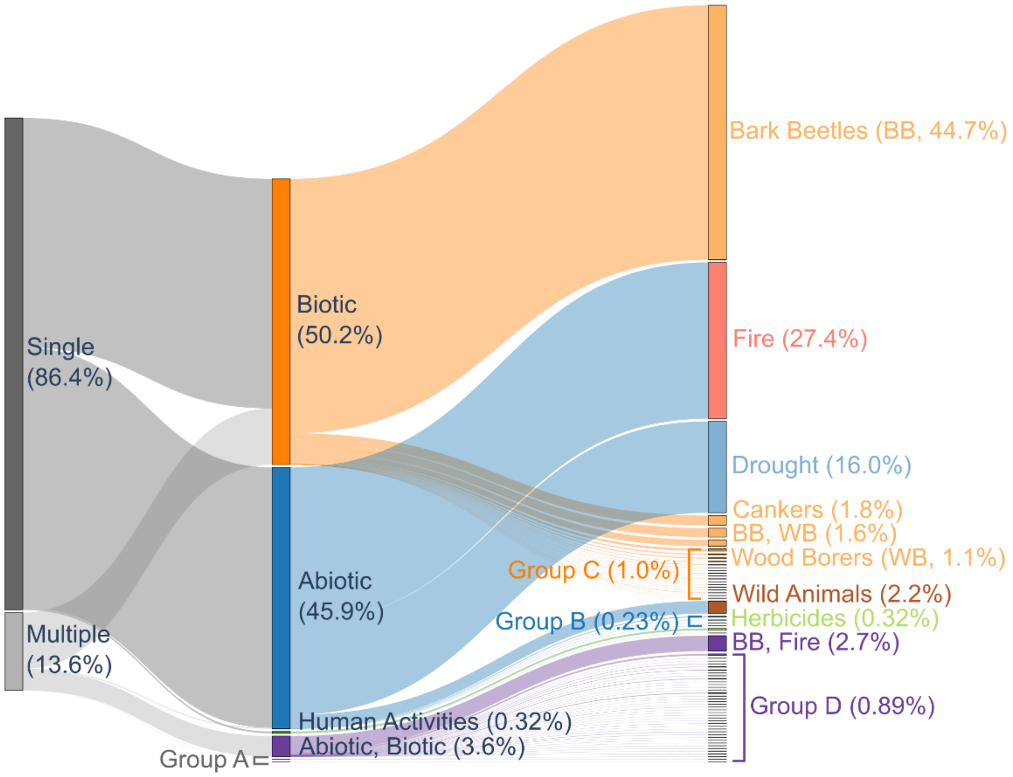

Supplementary Fig. 13: Percentage of impacted area from ADS database and fire perimeters from 2012-2020 by damage agent groups. Group A consists of two damage agent categories: Human Activities and Abiotic Agents (0.00013%) and Human Activities and Biotic Agents (0.00157%). The detailed information for Group B, C, and D is listed in Supplementary Table 10.

Supplementary Table 1: Accuracy assessment against tree-level ground observations.

Dataset	Abbr.	Year	Dead count	False negative	bias
SpeciesMap_Calibration_SouthBox_2020	SMSB2020	2020	32	0	0%
MCV_Validation_NorthBox_2020	MCVNB2020	2020	294	98	33.3%
Year 2020					16.7%
SpeciesMap_Validation_NorthBox_2019	SMNB2019	2019	73	29	39.7%
MCV_Calibration_ArrowCanopies_NorthBox_2018	MCVNB2018	2018	197	61	30.1%
SpeciesMap_Calibration_NorthBox_2016	SMNB2016	2016	98	19	19.4%
All years					24.7%

Supplementary Table 2: Tree mortality in California from 2012-2020 from ADS reports.

Year	Number of dead trees (million)	Impacted area (million hectares)	Survey area (million hectares)	Time of survey
2012	1.75	0.21	NA	June - September
2013	1.53	0.2	19.6	May - September
2014	3.3	0.37	17.8	April - September
2015	27.6	1.13	6.1	February, April, July - September
2016	62	NA	19	July - September
2017	27	NA	NA	August - November
2018	18	0.81	15	July - September
2019	15.1	0.89	16.6	June - August
2020	0.42*	NA	1.5	April - October
Sum	**156.7**			

* The flight was cancelled. The number was an estimation based on visual estimation from high-resolution imagery over 20 AOIs as shown in Supplementary Fig. 7. The associated time of survey refers to the dates of the imagery used. NA refers to no data available.

Supplementary Table 3: Lookup table for the abbreviations of 17 national forests in California.

Abbreviation	National forest name
ANF	Angeles National Forest
CNF	Cleveland National Forest
ENF	Eldorado National Forest
INF	Inyo National Forest
KNF	Klamath National Forest
LNF	Lassen National Forest
LPF	Los Padres National Forest
MNF	Mendocino National Forest
MDF	Modoc National Forest
PNF	Plumas National Forest
BDF	San Bernardino National Forest
SQF	Sequoia National Forest
SHF	Shasta-Trinity National Forest
SNF	Sierra National Forest
SRF	Six Rivers National Forest
STF	Stanislaus National Forest
TNF	Tahoe National Forest

Supplementary Table 4: Accuracy assessment for multi-year predictions against tree-level ground observations.

Pred. year	Bias (% underestimation)								
	Pred. year	Avg. of all years	SMNB 2016	MCVNB 2018	SMNB 2019	MCVNB 2020	SMSB 2020	DSSB 2021	DS 2023
2022	38.4	53.1	48.0	48.7	60.3	53.4	84.4	37.7	39.0
2020	16.7	24.7	19.4	31.0	39.7	33.3	0	-	-
2018	36.0	22.6	9.2	36.0	-	-	-	-	-
2016	34.7	34.7	34.7	-	-	-	-	-	-

Supplementary Table 5: Breakdown of tree height classes for all dead trees in DX2016 dataset.

Code	Common Name	Count	% Medium & Tall (>=15m)	% Short (<15m)	% Medium (15-30m)	% Tall (>30m)
PIPO	ponderosa pine	145	64.1	35.9	17.9	46.2
PILA	sugar pine	220	60.5	39.5	50.5	10
ABCO	white fir	1053	50.5	49.5	43.6	6.9
CADE	incense-cedar	1169	47.1	52.9	45.9	1.2
ALRH	white alder	5	40	60	40	0
QUKE	California black oak	198	26.3	73.7	26.3	0
UMCA	California-laurel	34	2.9	97.1	2.9	0
ABMA	California red fir	1	0	100	0	0
ACMA	bigleaf maple	2	0	100	0	0
CONU	Pacific dogwood	20	0	100	0	0
QUCH	canyon live oak	36	0	100	0	0
TOCA	California torreya (nutmeg)	26	0	100	0	0

Supplementary Table 6: List of ancillary datasets.

Datasets	Time frame	Format	Spatial resolution	References
California state boundary	2016	Shapefile		[61]
U.S. Environmental Protection Agency Level III ecoregions in California	2013	Shapefile: polygon		[62]
Woodland for California	2011	Shapefile: polygon		[60]
Cities and urban areas	2013	Shapefile: polygon		[65,66]
Inland water bodies	2004	Shapefile: polygon		[67]
USA National Land Cover Database	2019	.tif	30 m	[63]
ESA WorldCover 2020	2020	.tif	10 m	[64]
Forest Type Groups of the Continental United States	2014-2018	.tif	30 m	[44]
Forest Type of the Continental United States	2002-2003	.tif	250 m	[44]
US Forest Service Administrative Forest Boundaries		Shapefile: polygon		[68]
Aerial Detection Survey (ADS)	2012-2020	Shapefile: polygon and point		[10]
Global Forest Change v1.9	2000-2020	.tif	30 m	[27]
F3 snag density	2021	.tif	30 m	[70]
Fire perimeters	2012-2020	Shapefile: polygon		[46]
Fuel Disturbance	2012-2020	.tif	30 m	[69]
Trees Per Acre	2011	.tif	240 m	[43]

Supplementary Table 7: Classification of height classes based on DBH and species-specific allometry equations suggested by ref.[57].

Code	Common Name	Scientific Name	DBH (cm) for Short class (<15m)	DBH (cm) for Medium class (15-30m)	DBH (cm) for Tall class (>30m)
PIPO	ponderosa pine	Pinus ponderosa	<20	20-40	40-150
PILA	sugar pine	Pinus lambertiana	<25	25-55	55-175
ABCO	white fir	Abies concolor	<20	20-50	50-150
CADE	incense-cedar	Calocedrus decurrens	<30	30-65	65-145
ALRH	white alder	Alnus rhombifolia	<15	15-55	-
QUKE	California black oak	Quercus kelloggii	<25	25-75	75-135
UMCA	California-laurel	Umbellularia californica	<25	25-30	-
ABMA	California red fir	Abies magnifica	<25		
ACMA	bigleaf maple	Acer macrophyllum	<15	15-40	-
CONU	Pacific dogwood	Cornus nuttallii	<35	-	-
TOCA	California torreya (nutmeg)	Torreya californica	10-50	-	-

Supplementary Table 8: Regrouping of damage agents from ADS and fire perimeters from 2012-2020.

Level 1	Level 2	Level 3 (ADS Category)	Level 4 (ADS Label)
Biotic	Insects	Bark Beetles	California fivespined ips, Douglas-fir beetle, Douglas-fir engraver, Jeffrey pine beetle, cedar & cypress bark beetles, fir engraver, fir root bark beetle, ips engraver beetles, mountain pine beetle, pine engraver, pinyon ips, red turpentine beetle, spruce beetle, twig beetles, unknown bark beetle, western cedar bark beetle, western pine beetle
		Wood Borers	Boring Insects, California flatheaded borer, flatheaded fir borer, goldspotted oak borer, polyphagous shot hole borer, unknown wood borer
	Diseases	Cankers	Cytospora canker of fir, other canker agent, known (code pending), pitch canker of pines (Gibberella circinata also known as Fusarium circinatum), sudden oak death, thousand cankers disease
		Rusts	comandra blister rust, western gall rust, white pine blister rust
		Foliage and Shoot Diseases	Diplodia blight, Dothistroma needle blight (D. septosporum), Elytroderma needle blight, Foliage diseases, Marssonina blight, anthracnose, needlecast, sycamore anthracnose, unknown foliage or shoot disease
		Root Diseases / Decay	Port-Orford-cedar root disease, Root/Butt Diseases, black stain root disease, canker rot of oak, unknown root disease or decay
		Parasitic Higher Plants	true mistletoe
	Defoliators	Defoliators	California oakworm, Douglas-fir tussock moth, Gelechiid moths/needleminers, Modoc budworm, alder flea beetle, fall webworm, forest tent caterpillar, fruittree leafroller, lodgepole needleminer, needleminers, pinyon sawfly, satin moth, sawflies, unknown defoliator, western spruce budworm
		Sap Feeders	black pineleaf scale, pinyon needle scale, spruce aphid
Human Activities	Human Activities	Herbicides	herbicides
		Other Human Activities	unknown human activities, mechanical
Abiotic	Drought	Drought	drought
	Fire	Fire	fire
	Wild Animals	Wild Animals	bears, squirrels, unknown wild animal
	Other Abiotic Agents	Other Abiotic Agents	Abiotic Damage, avalanche, flooding-high water, frost, lightning,mud-land slide, nutrient imbalances, snow-ice, wind-tornado, wind-tornado/hurricane

Supplementary Table 9: Percentage of total count of dead trees detected for each damage agent category.

Level 1	Level 3 (ADS Category)	Percentage (%)
Abiotic	Fire	27.2
	Drought	16.5
	Wild Animals	1.2
Biotic	Bark Beetles	42.5
	Cankers	1.5
	Bark Beetles,Wood Borers	0.89
	Wood Borers	0.89
Human Activities	Herbicides	0.33
	Other Human Activities	0.000019
Abiotic,Biotic	Bark Beetles,Fire	7.1
Group B		
Abiotic	Drought,Fire	0.16
	Other Abiotic Agents	0.03
	Wild Animals,Fire	0.012
	Other Abiotic Agents,Fire	0.004
Group C		
Biotic	Defoliators	0.17
	Bark Beetles,Cankers	0.16
	Multi-Agent "Complexes"	0.087
	Foliage and Shoot Diseases	0.058
	Bark Beetles,Multi-Agent "Complexes"	0.034
	Sap Feeders	0.02
	Rusts	0.017
	Bark Beetles,Defoliators	0.013
	Bark Beetles,Rusts	0.0071
	Cankers,Wood Borers	0.0035
	Root Diseases / Decay	0.00058
	Multi-Agent "Complexes",Wood Borers	0.00049
	Bark Beetles,Foliage and Shoot Diseases	0.00022
	Parasitic Higher Plants	0.00019
	Root Diseases / Decay,Wood Borers	0.000093
	Cankers,Foliage and Shoot Diseases	0.000055
	Cankers,Multi-Agent "Complexes"	0.000032
Group D		
Abiotic,Biotic	Wood Borers,Fire	0.24
	Cankers,Fire	0.23
	Bark Beetles,Wood Borers,Fire	0.2
	Drought, Bark Beetles	0.11
	Bark Beetles,Cankers,Fire	0.1
	Drought,Multi-Agent "Complexes"	0.057
	Multi-Agent "Complexes",Fire	0.036
	Drought,Multi-Agent "Complexes",Fire	0.028
	Defoliators,Fire	0.025
	Drought,Wood Borers	0.024
	Drought,Bark Beetles,Multi-Agent "Complexes"	0.013
	Bark Beetles,Multi-Agent "Complexes",Fire	0.0095
	Drought,Bark Beetles,Fire	0.0081

	Drought,Cankers,Fire	0.0047
	Drought,Wood Borers,Fire	0.0045
	Drought,Bark Beetles,Wood Borers,Fire	0.0044
	Drought,Cankers	0.004
	Drought,Bark Beetles,Cankers	0.0023
	Rusts,Fire	0.0014
	Drought,Bark Beetles,Wood Borers	0.0013
	Other Abiotic Agents,Rusts	0.0012
	Foliage and Shoot Diseases,Fire	0.00087
	Bark Beetles,Defoliators,Fire	0.0008
	Bark Beetles,Wild Animals	0.00062
	Wild Animals,Wood Borers	0.0005
	Bark Beetles,Wild Animals,Fire	0.0004
	Root Diseases / Decay,Wild Animals	0.00039
	Cankers,Other Abiotic Agents	0.00029
	Multi-Agent "Complexes",Wood Borers,Fire	0.00028
	Bark Beetles,Rusts,Fire	0.00025
	Drought,Defoliators,Other Abiotic Agents	0.000093
	Cankers,Wild Animals	0.000076
	Drought,Bark Beetles,Multi-Agent "Complexes",Fire	0.000051
	Cankers,Multi-Agent "Complexes",Fire	0.000029
	Bark Beetles,Rusts,Wild Animals	0.000028
	Drought,Defoliators	0.0000055
Biotic,Human Activities	Herbicides,Wood Borers	0.00014
Abiotic,Human Activities	Herbicides,Fire	0.00086
	Herbicides,Wild Animals	0.00024

Supplementary Table 10: Percentage of impacted area recorded in ADS and fire polygons from 2012-2020 for each damage agent category.

Level 1	Level 3 (ADS Category)	Percentage (%)
Abiotic	Fire	27.4
	Drought	16.0
	Wild Animals	2.2
Biotic	Bark Beetles	44.7
	Cankers	1.8
	Bark Beetles, Wood Borers	1.6
	Wood Borers	1.1
Human Activities	Herbicides	0.32
	Other Human Activities	0.000025
Abiotic,Biotic	Bark Beetles, Fire	2.7
Group B		
Abiotic	Drought, Fire	0.16
	Other Abiotic Agents	0.063
	Other Abiotic Agents, Fire	0.0047
	Wild Animals, Fire	0.0037
Group C		
Biotic	Defoliators	0.37
	Bark Beetles, Cankers	0.20
	Multi-Agent "Complexes"	0.17
	Foliage and Shoot Diseases	0.10
	Bark Beetles, Multi-Agent "Complexes"	0.081
	Sap Feeders	0.059
	Rusts	0.025
	Bark Beetles, Defoliators	0.016
	Bark Beetles, Rusts	0.014
	Cankers, Wood Borers	0.006
	Multi-Agent "Complexes", Wood Borers	0.0020
	Parasitic Higher Plants	0.00082
	Root Diseases / Decay	0.00068
	Bark Beetles, Foliage and Shoot Diseases	0.00060
	Cankers, Foliage and Shoot Diseases	0.00041
	Cankers, Multi-Agent "Complexes"	0.00039
	Root Diseases / Decay, Wood Borers	0.000043
Group D		
Abiotic,Biotic	Bark Beetles, Drought	0.29
	Wood Borers, Fire	0.12
	Drought, Multi-Agent "Complexes"	0.10
	Cankers, Fire	0.089
	Bark Beetles, Wood Borers, Fire	0.074
	Drought, Wood Borers	0.040
	Bark Beetles, Cankers, Fire	0.036
	Multi-Agent "Complexes", Fire	0.034
	Bark Beetles, Drought, Multi-Agent "Complexes"	0.023
	Drought,Multi-Agent "Complexes", Fire	0.020
	Drought, Wood Borers, Fire	0.010
	Bark Beetles, Drought, Fire	0.010
	Cankers, Drought	0.010

	Defoliators, Fire	0.0070
	Bark Beetles, Multi-Agent "Complexes", Fire	0.0068
	Foliage and Shoot Diseases, Fire	0.0031
	Other Abiotic Agents, Rusts	0.0026
	Bark Beetles, Drought, Wood Borers	0.0025
	Bark Beetles, Cankers, Drought	0.0020
	Wild Animals, Wood Borers	0.0012
	Bark Beetles, Drought, Wood Borers, Fire	0.0012
	Bark Beetles, Wild Animals	0.0012
	Cankers, Drought, Fire	0.00089
	Bark Beetles, Defoliators, Fire	0.00080
	Bark Beetles, Rusts, Fire	0.00077
	Root Diseases / Decay, Wild Animals	0.00073
	Bark Beetles, Rusts, Wild Animals	0.00070
	Rusts, Fire	0.00069
	Defoliators, Drought, Other Abiotic Agents	0.00064
	Cankers, Other Abiotic Agents	0.00056
	Multi-Agent "Complexes", Wood Borers, Fire	0.00046
	Defoliators, Drought	0.00032
	Bark Beetles, Drought, Multi-Agent "Complexes", Fire	0.00025
	Cankers, Wild Animals	0.00016
	Bark Beetles, Wild Animals, Fire	0.00012
	Cankers, Multi-Agent "Complexes", Fire	0.0000087
Biotic,Human Activities	Herbicides, Wood Borers	0.00013
Abiotic,Human Activities	Herbicides, Wild Animals	0.00095
	Herbicides, Fire	0.00062
