# Supplementary material for: Scattered tree death contributes to substantial forest loss in California
Source: Nat Commun. 2024 Jan 20;15:641. doi: 10.1038/s41467-024-44991-z (PMC10799937; doi:10.1038/s41467-024-44991-z)
Supplement: Supplementary file 3 — Reporting Summary [file 41467_2024_44991_MOESM3_ESM.pdf]

nature portfolio

Corresponding author(s): Yan Cheng, Stephanie Horion

Last updated by author(s): Dec 16, 2023

Reporting Summary

Nature Portfolio wishes to improve the reproducibility of the work that we publish. This form provides structure for consistency and transparency in reporting. For further information on Nature Portfolio policies, see our Editorial Policies and the Editorial Policy Checklist.

Statistics

For all statistical analyses, confirm that the following items are present in the figure legend, table legend, main text, or Methods section.

n/a	Confirmed	
☐	☒	The exact sample size (*n*) for each experimental group/condition, given as a discrete number and unit of measurement
☐	☒	A statement on whether measurements were taken from distinct samples or whether the same sample was measured repeatedly
☐	☒	The statistical test(s) used AND whether they are one- or two-sided<br>*Only common tests should be described solely by name; describe more complex techniques in the Methods section.*
☒	☐	A description of all covariates tested
☐	☒	A description of any assumptions or corrections, such as tests of normality and adjustment for multiple comparisons
☐	☒	A full description of the statistical parameters including central tendency (e.g. means) or other basic estimates (e.g. regression coefficient) AND variation (e.g. standard deviation) or associated estimates of uncertainty (e.g. confidence intervals)
☒	☐	For null hypothesis testing, the test statistic (e.g. *F*, *t*, *r*) with confidence intervals, effect sizes, degrees of freedom and *P* value noted<br>*Give P values as exact values whenever suitable.*
☒	☐	For Bayesian analysis, information on the choice of priors and Markov chain Monte Carlo settings
☒	☐	For hierarchical and complex designs, identification of the appropriate level for tests and full reporting of outcomes
☒	☐	Estimates of effect sizes (e.g. Cohen's *d*, Pearson's *r*), indicating how they were calculated

Our web collection on statistics for biologists contains articles on many of the points above.

Software and code

Policy information about availability of computer code

Data collection	We used publicly available aerial images from National Agriculture Imagery Program (NAIP). NAIP aerial images were downloaded from Google Earth Engine (GEE), which is a free platform. The code used for downloading NAIP from GEE was written in Python (3.9). The code can be accessed at https://doi.org/10.6084/m9.figshare.23723388
Data analysis	All analyses were conducted using open-source software, i.e., Python (3.9) and QGIS (3.24). The deep learning algorithm was written in Python (3.9) using PyTorch. The post-processing analyses codes were written in Python (3.9). All codes can be accessed at https://doi.org/10.6084/m9.figshare.23723388

For manuscripts utilizing custom algorithms or software that are central to the research but not yet described in published literature, software must be made available to editors and reviewers. We strongly encourage code deposition in a community repository (e.g. GitHub). See the Nature Portfolio guidelines for submitting code & software for further information.

Data

Policy information about availability of data

All manuscripts must include a data availability statement. This statement should provide the following information, where applicable:

- Accession codes, unique identifiers, or web links for publicly available datasets
- A description of any restrictions on data availability
- For clinical datasets or third party data, please ensure that the statement adheres to our policy

NAIP images are freely available on Google Earth Engine (https://developers.google.com/earth-engine/datasets/catalog/USDA_NAIP_DOQQ). The sources of all ancillary datasets are listed in Supplementary Table 6. Derived products, i.e., dead tree count per ha (100 m), median dead crown size per ha (100 m), percentage of dead canopy area per ha (100 m), percentage of brown-stage mortality per ha (100 m), eccentricity map (500 m), and percentage of tree mortality (240 m), are freely accessible at ref.78. Field observations DX2016 and MCVNB2018 are available from ref.13,57 and ref.77 respectively. Field observations (datasets SMNB2016, SMNB2019, MCVNB2020, SMSB2020, and DSSB2021) are available from A.D.. and DS2023 is available from A.D. and Y.C..

Research involving human participants, their data, or biological material

Policy information about studies with human participants or human data. See also policy information about sex, gender (identity/presentation), and sexual orientation and race, ethnicity and racism.

Reporting on sex and gender	NA
Reporting on race, ethnicity, or other socially relevant groupings	NA
Population characteristics	NA
Recruitment	NA
Ethics oversight	NA

Note that full information on the approval of the study protocol must also be provided in the manuscript.

Field-specific reporting

Please select the one below that is the best fit for your research. If you are not sure, read the appropriate sections before making your selection.

☐ Life sciences ☐ Behavioural & social sciences ☒ Ecological, evolutionary & environmental sciences

For a reference copy of the document with all sections, see nature.com/documents/nr-reporting-summary-flat.pdf

Ecological, evolutionary & environmental sciences study design

All studies must disclose on these points even when the disclosure is negative.

Study description	This study mapped and characterized tree mortality in California at the tree level from 7,645 tiles of NAIP aerial images at 60 cm resolution collected in 2020. We manually digitized ~27,000 dead tree crowns spanning a wide range of landscapes in California and trained a deep learning model to automatically segment individual dead trees from NAIP aerial images. We applied the watershed algorithm to separate clumped dead tree crowns. We evaluated the accuracy against ground observations of dead trees at the plot and the tree level. We compared the individual dead tree map to global forest loss data derived from Landsat images at 30 m resolution. We assessed tree mortality in California by forest type groups and damage agents supported by ancillary datasets. Going beyond the localization of individual dead trees, we extracted the size and shape of dead tree crowns and classified mortality stage (red vs. gray stage) based on the color of individual dead tree crowns. We then aggregated the dead tree crown size, dead tree count, and percentage of red-stage dead trees within hectare grids. These metrics were used to map spots with high ecological interests such as areas with a high density of standing deadwood and/or with recent mortality. Lastly, we applied the model trained for 2020 directly to NAIP images acquired in adjacent years to map multi-year tree mortality over a spatial subset of the study area and evaluated the accuracy against ground observations.
Research sample	This study presented a state-wide mapping and characterization of tree mortality at the individual tree level, where the location, dead crown size, and mortality stage of all identified dead trees were mapped over 27.8 million hectares of vegetated area (excl. agriculture areas) in California.
Sampling strategy	We did not apply a specific sampling strategy. Dead trees for all species over vegetated areas (excl. agriculture areas) were included in this study. However, due to the constrain of the spatial resolution of NAIP images, dead trees with crowns smaller than 0.36 m2 were excluded as they are not visible from NAIP aerial images.
Data collection	We used publicly available aerial images from the National Agriculture Imagery Program (NAIP) administered through USDA Farm

Data collection

Production and Conservation - Business Center, Geospatial Enterprise Operations. NAIP data consists of sub-meter to meter resolution optical images that have been acquired every one to two years during the growing season at pan-US scales since 2003 . In this study, we downloaded 7,645 NAIP tiles (>3.4TB) acquired in 2020 from Google Earth Engine to have complete coverage over vegetated areas (excl. agriculture areas) in California. These images were taken from the middle of April to early August. These images have 60 cm spatial resolution and four spectral bands, i.e., red (619-651nm), green (525-585nm), blue (435-495nm), and near-infrared (808-882nm) bands. The georeferencing accuracy (RMSE) was reported as 6 meters. The ground observations of dead trees were collected between 2016 and 2023 in summer in the southern Sierra Nevada. In DX2016 dataset, the number of dead trees >= 1.35 m in height and >=2 cm DBH within a 0.1 ha-large plot were recorded. A total of 75 plots were used in this study, which consists of 2,921 dead trees with 327 dead tree >=40 DBH. In the other datasets collected in 2016, 2018, 2019, 2020, 2021, and 2023, the locations of 971 dead trees >=40cm in DBH were recorded.

Timing and spatial scale

The NAIP images were taken from the middle of April to early August in 2020 covering 27.8 million hectares of vegetated area (excl. agriculture and desert areas) in California (extent: -124.41,42.01 : -114.13,32.53). The ground observations were collected from 2016, 2018, 2019, 2020, 2021, and 2023 in summer and are located in the southern Sierra Nevada (extent: -118.9673,36.8094 : -118.6021,36.4168).

Data exclusions

In Central Valley and Mojave Desert, NAIP aerial images outside the woodland footprint were not included in this study given that these areas have <5% tree cover. In addition, water bodies and cities were masked out using ancillary datasets.

Reproducibility

We conduced the analyses for 3 times which led to the same results.

Randomization

NA. The study covers all 27.8 million hectares of vegetated area (excl. agriculture and desert areas) in California.

Blinding

NA. The study covers all 27.8 million hectares of vegetated area (excl. agriculture and desert areas) in California.

Did the study involve field work? ☒ Yes ☐ No

Field work, collection and transport

Field conditions

Fieldwork were conducted in summer 2016, 2018, 2019, 2020, 2021, and 2023.

Location

The ground observations were collected in the southern Sierra Nevada (extent: -118.9673,36.8094 : -118.6021,36.4168).

Access & import/export

NA. No physical samples were collected and all the observations were recorded digitally.

Disturbance

The coordinates of dead trees observed but located in non-accessible areas were not recorded.

Reporting for specific materials, systems and methods

We require information from authors about some types of materials, experimental systems and methods used in many studies. Here, indicate whether each material, system or method listed is relevant to your study. If you are not sure if a list item applies to your research, read the appropriate section before selecting a response.

Materials & experimental systems

n/a	Involved in the study	
☒	☐	Antibodies
☒	☐	Eukaryotic cell lines
☒	☐	Palaeontology and archaeology
☒	☐	Animals and other organisms
☒	☐	Clinical data
☒	☐	Dual use research of concern
☒	☐	Plants

Methods

n/a	Involved in the study	
☒	☐	ChIP-seq
☒	☐	Flow cytometry
☒	☐	MRI-based neuroimaging
